# Non-Linear Optical Properties of Er^3+^–Yb^3+^-Doped NaGdF_4_ Nanostructured Glass–Ceramics

**DOI:** 10.3390/nano10071425

**Published:** 2020-07-21

**Authors:** José J. Velázquez, Giulio Gorni, Rolindes Balda, Joaquin Fernández, Laura Pascual, Alicia Durán, Maria J. Pascual

**Affiliations:** 1 Centre for Functional and Surface Functionalized Glass (FunGlass), Alexander Dubček University of Trenčín, 911 50 Trenčín, Slovakia; 2 Instituto de Cerámica y Vidrio, ICV-CSIC, 28049 Madrid, Spain; ggorni@icv.csic.es (G.G.); aduran@icv.csic.es (A.D.); mpascual@icv.csic.es (M.J.P.); 3 Applied Physic Department I, Superior School of Engineering, Basque Country University, 48013 Bilbao, Spain; rolindes.balda@ehu.eus; 4 Materials Physics Center CSIC-UPV/EHU, 20018 San Sebastian, Spain; 5 Donostia International Physics Center (DIPC), 20018 San Sebastian, Spain; xuaco@dipc.org; 6 Instituto de Catálisis y Petroleoquímica-CSIC, 28049 Madrid, Spain; laura.pascual@icp.csic.es

**Keywords:** oxyfluoride glass–ceramic, crystallization, rare-earth, nonlinear optical properties

## Abstract

Transparent oxyfluoride glass–ceramics containing NaGdF_4_ nanocrystals were prepared by melt-quenching and doped with Er^3+^ (0.5 mol%) and different amounts of Yb^3+^ (0–2 mol%). The selected dopant concentration the crystallization thermal treatments were chosen to obtain the most efficient visible up-conversion emissions, together with near infrared emissions. The crystal size increased with dopant content and treatment time. NaGdF_4_ NCs with a size ranging 9–30 nm were obtained after heat treatments at T_g_ + 20–80 °C as confirmed by X-ray diffraction and high-resolution transmission electron microscopy. Energy dispersive X-ray analysis shows the incorporation of rare earth ions into the NaGdF_4_ nanocrystals. Near-infrared emission spectra, together with the up-conversion emissions were measured. The optical characterization of the glass–ceramics clearly shows that Er^3+^ and Yb^3+^ ions are incorporated in the crystalline phase. Moreover, visible up-conversion emissions could be tuned by controlling the nanocrystals size through appropriated heat treatment, making possible a correlation between structural and optical properties.

## 1. Introduction

The develop of transparent rare-earth (RE^3+^)-doped oxyfluoride glass–ceramics (GCs) in which the nanocrystals (NCs) are homogeneously distributed in a glass matrix have gained increasing attention for their application in more efficient photonic devices for energy and optics. This kind of materials combines the incorporation of active rare-earth (RE^3+^) ions into low phonon energy fluoride crystals (300–450 cm^−1^) and the good thermal stability and high mechanical and chemical properties of an aluminosilicate glass matrix [1,2,3]. Moreover, the different synthesis methods to obtain these materials (melt-quenching, sol–gel, hydrothermal, etc) together with the combination of host matrix, RE doping and NC size allow the luminescent emission to be properly tuned, thus opening the way to different promising applications such as optical fibers, solid-state lasers, three-dimensional full-color displays and solar cells among others [4,5,6,7,8].

Oxyfluoride GCs can be obtained after careful control of the crystallization process of the precursor glass using adequate heat treatments. In that case, homogeneously dispersed nanocrystalline particles grow following a diffusion-controlled process, with NCs size ranging from 10 to 50 nm. The importance of keeping the nanometric size of the crystals is essential to have high quality transparent optical materials with reduced Rayleigh scattering [9,10]. Additionally, RE^3+^ ions can be efficiently incorporated in the fluoride NCs [11,12,13,14,15]. Thus, accurate control of the crystallization process is required to increase the photoluminescence efficiency and thus, the optical quality of these materials. The crystallization mechanism of different fluoride phases, such as LaF_3_, NaLaF_4_, KaLaF_4_ among others [7,16,17,18] in aluminosilicate glasses occurs via phase separation with the formation of droplets enriched in Si-, F-, La-, Y- or Lu-. During the crystallization process, the glass matrix gets depleted of crystal formers and a viscous barrier made of network formers, mainly SiO_2_, surround the NCs. The former barrier avoids further crystal growth, limiting the NCs size to the nanometric scale.

On the other hand, the selection of a suitable fluoride matrix for each specific application also plays a relevant role. In particular, polymorph crystalline phases such as NaREF_4_, with Gd, Y, La or Lu, as RE, have been reported to be suitable luminescence upconverter matrix for Ln^3+^. Particularly, in the case of hexagonal NaREF_4_ phases, the low symmetry positions of the dopant ions increase the transition probabilities of partially forbidden intra-configurational 4f–4f transitions [17]. Taking into account this consideration, the NaGdF_4_ matrix has been reported as one of the best converter hosts for Ln^3+^ ions, such as Yb^3+^–RE^3+^ pairs [19,20,21,22,23], among different polymorphous NaREF_4_ matrices. The presence of two NaGdF_4_ crystalline phases, such as the cubic (α) and hexagonal (β), also allows comparing the relative efficiency related to optical applications. Different studies published in past years indicate that the luminescence efficiency of RE^3+^-doped ions in this host seems to be greater in the β-NaGdF_4_ respecting to the α-phase [24,25,26]. Moreover, Gd ions can be replaced by RE ions when the material is doped. As a result, optical properties are considerably improved respecting to the precursor glass. Several articles are focused on the preparation and optical properties of the β-NaGdF_4_ containing GCs. In particular, Andreas Herrmann et al. analyzed the precipitation of Sm^3+^-doped NaGdF_4_ NCs in glass and its effect on the optical properties when using the emissions of Sm^3+^ ions as a structural probe. Herrmann determined that a higher Na/Gd ratio favors the crystallization of the β-NaGdF_4_ phase respecting to the cubic phase and as a result, the optical properties of Sm^3+^ ions are finally enhanced [27].

The structural-optical properties relationship were also investigated by others authors showing that the up-conversion (UC) efficiency increases in Er^3+^–Yb^3+^ and Pr^3+^–Yb^3+^ co-doped NaGdF_4_ GCs [28,29,30]. They observed changes in optical features (Stark splitting, full width at half maximum, etc.) of the luminescence. Moreover, they found that the intensity is several times stronger for GCs concerning those obtained for the precursor glass due to the incorporation of RE^3+^ ions into the nanocrystalline phase.

In this study, we report the preparation, structural and optical properties of Er^3+^-doped and Er^3+^–Yb^3+^-codoped NaGdF_4_ GCs mainly focusing in the near-infrared (NIR) and up-conversion (UC) emissions due to the possibility to increase the efficiency of the energy transfer processes. In particular, the effect of temperature and the RE^3+^ ions incorporation on the crystallization and crystal growth was analyzed. The UC dependence emission on the excitation as well as the chromaticity dependence on the temperature was studied for different samples, giving us the capability to tune the emitting color by controlling the NCs size resulting in an available color gamut.

## 2. Materials and Methods

### 2.1. Materials Preparation

The precursor glasses with composition 70SiO_2_-7Al_2_O_3_-8Na_2_O-8K_2_O-7GdF_3_-0.5ErF_3_–xYbF_3_ (70Si7Gd, x = 0 mol% and 2 mol%) were prepared by the melt-quenching method. The raw materials of reagent grade that were used are SiO_2_ (Saint-Gobain, France, 99.6%), Al_2_O_3_ (Panreac, Barcelona, Spain), Na_2_CO_3_ (Sigma-Aldrich, St. Louis, MO, USA, 99.5%), K_2_CO_3_ (Scharlau, Barcelona, Spain 99.99%), GdF_3_ (Alfa Aesar, Haverhill, MA, USA, 99.9%), ErF_3_ (Alfa Aesar, 99.99%) and YbF_3_ (Alfa Aesar, 99.999%). The batches were mixed by milling for 3 h. After that they were calcined at 1200 °C for 2 h, melted at 1600 °C for 1 h and then fast quenched onto a brass mold. The melting process was repeated twice to increase the glass homogeneity and then the glasses were annealed at 515 °C for 30 min for stress relaxation. Bulk glass specimens were heat-treated at 550, 580, 600 and 620 °C for 5 ÷ 120 h, using a heating rate of 10 °C/min to get the corresponding GCs. Polished glass and GC sheets (1 cm × 1 cm × 2 mm) were prepared for optical characterization.

### 2.2. Thermal and Structural Characterization

Thermal parameters, such as glass transition temperature (T_g_), softening point (T_d_) and thermal expansion coefficient (α) were determined by dilatometry using a Netzsch Gerätebau dilatometer, model 402 PC/1 (NETZSCH GmbH & Co. Holding, Selb, Germany) with a heating rate of 5 °C/min in air; the estimated error of T_g_ is ± 2 °C.

X-ray diffraction (XRD) measurements were done with an X-ray diffractometer D8 ADVANCE (Bruker, Billerica, MA, USA) equipped with a Lynx Eye detector. The glass and GCs pieces were milled to a particle-size less than 60 μm. The diffractograms were collected using monochromatic CuKα_1_ radiation (1.54056 Å) in the 10 ≤ 2θ ≤ 70° range with steps of 0.02° and 1-s acquisition per step. The crystal phase was analyzed with the software EVA Difrac PLUS (V2, Karlsruhe, Germany). The crystallized size, *ϕ* was determined using the Scherrer equation [31]:

(1) $$\phi =\frac{0.94\cdot \lambda }{\cos\theta \;\cdot \sqrt{B^{2}-B_{i}^{2}}}$$

where *θ* is the angle of the diffraction maximum, *B* is the FWHM, *B_i_* is the instrumental broadening and *λ* is the wavelength. The *θ* and *B* parameters were obtained by fitting the peaks to pseudo-Voight function.

TEM samples of glasses and GCs were prepared using sieved powders of size <63 µm. High-resolution electron microscopy (HR-TEM), including scanning transmission microscopy-high angle annular dark field (STEM-HAADF) and X-ray energy dispersive spectroscopy (EDXS), were recorded on a JEOL 2100 field emission gun transmission electron microscope (Akishima, Tokyo, Japan) operating at 200 kV and providing a point-resolution of 0.19 nm. The TEM is equipped with an EDXS energy dispersive X-ray spectrometer (INCA x-sight, Oxford Instruments). EDX analysis was performed in STEM mode, with a probe size of ca. 1 nm. Samples were prepared by dispersing the powder, obtained by grinding the glass and GC pieces using ethanol with ultrasonic agitation; a few droplets of the suspension were deposited on a perforated copper–carbon grid and the solvent was finally removed by drying under a UV lamp.

### 2.3. Optical Characterization

Ultraviolet-visible (UV-Vis) absorption spectra of polished glass and GCs sheets were recorded in the 800–1100 nm range using a Lamba 900 PerkinElmer double beam spectrophotometer (Waltham, MA, USA). Optical properties in the form of steady-state emission spectra were made with a Ti:sapphire ring laser (0.4 cm^−1^ linewidths). The fluorescence was analyzed with a 0.25 monochromator and the signal was detected by Hamamatsu R636 and H10330A-75 photomultipliers (Hamamatsu, Shizuoka, Japan) and finally amplified by a standard lock-in technique. All measurements were taken at room temperature.

## 3. Results and Discussion

**Figure 1:**
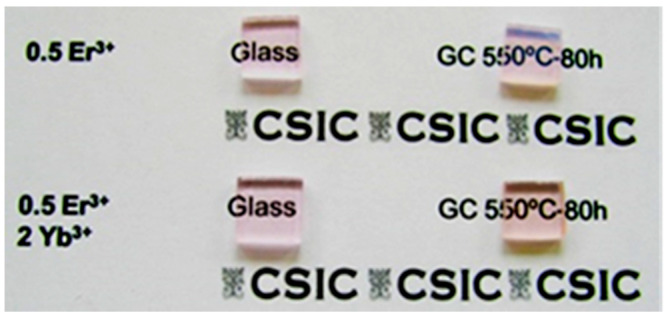
Transparent samples of 0.5Er^3+^-doped and 0.5Er^3+^–2Yb^3+^-codoped 70Si7Gd glass and glass–ceramics (GCs) heat-treated at 550 °C-80 h.

Precursor glasses and respective GCs were obtained for each composition after treatment at 550–620 °C for 80–120 h. Transparency of these GCs remained after heat treatment at higher temperatures up to 620 °C (not shown). As an example, Figure 1 shows some glass and GC samples used for optical characterization.

### 3.1. Thermal and Structural Properties

The thermal behavior of precursor glasses was studied using dilatometry analysis. This technique permits parameters such as the glass transition temperature (T_g_), dilatometric softening temperature (T_d_) and thermal expansion coefficient T_EC_(α) to be obtained Table 1 summarizes the results of dilatometry measurements as a function of the RE doping. As it can be observed, T_g_ increases from 510 to 546 °C with the increment of the dopant content, which is in agreement with previous results obtained by the authors [30] in a similar composition, but doped with Pr^3+^ and Pr^3+^-Yb^3+^. It means that this behavior depends mainly on the dopant content. T_d_ shows a similar behavior, increasing its value from 590 to 648 °C. For this reason, the optimum range of heat treatment temperatures was selected as T_g_ + (20 ÷ 100) °C. On the other hand, α remains almost constant. This behavior confirms that the phase separation is favored by the dopant incorporation in the corresponding oxyfluoride precursor glasses, leading to an increase of the glass viscosity. A similar effect was previously observed by Sroda and A. de Pablos-Martin et al. [7,32].

**Figure 2:**
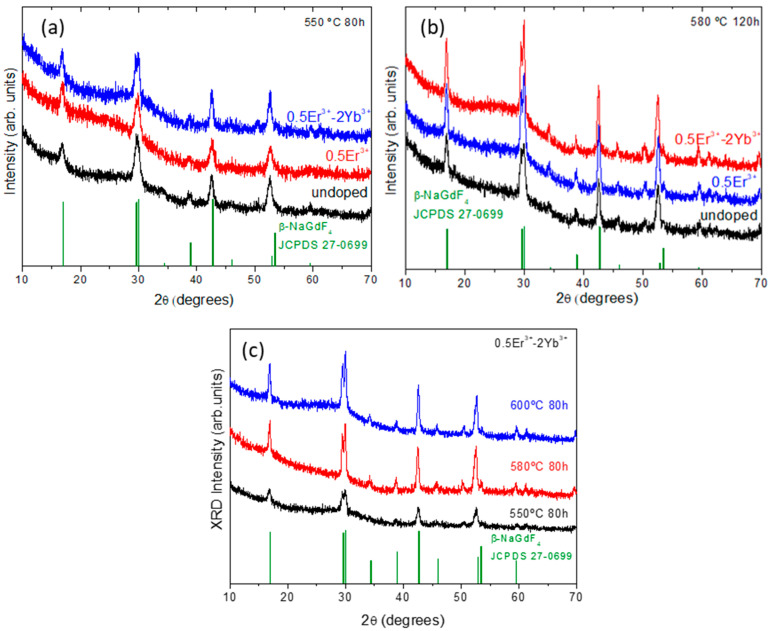
(**a**) XRD patterns for undoped, Er^3+^-doped and Er^3+^–Yb^3+^-codoped 70Si7Gd GCs treated at 550 °C-80 h and (**b**) 580 °C-120 h; (**c**) XRD patterns of 0.5Er–2Yb-codoped 70Si7Gd GCs at different treatment temperatures for 80 h.

A structural characterization was performed by XRD to analyze the effect of the treatment temperature and dopant incorporation in NaGdF_4_ NCs. Figure 2a,b show XRD diffractograms of undoped, Er^3+^-doped and Er^3+^–Yb^3+^-codoped samples treated between 550-80 h and 580 °C-120 h whereas Figure 2c shows the effects of the temperature on the crystal growth of Er^3+^–Yb^3+^-codoped samples. These diffractograms show typical peaks of β-NaGdF_4_ (JCPDS 27–0699) crystalline phase (Figure 2). The XRD patterns of glasses (not shown) did not reveal the existence of any crystalline phase. The peaks become narrower and more intense with increasing temperature, indicating that the NCs growth is favored by increasing the temperature. The same behavior is observed increasing the dopant concentration up to 2.5 mol%. Beyond such value, there is a clear decrease in the intensity and the peaks become broader. Similar conclusions were drawn in a previous study in which the XRD peaks intensity increased for doping level up to 3 mol% but no crystalline phases were detected for doping concentration higher than 4.5 mol% [30,33]. A possible explanation is that when the doping level becomes as high as the content of crystal formers, such as Gd^3+^and Lu^3+^, can strongly affect the crystallization process [33,34].

The mean size of NCs was calculated by using the Scherrer equation (Equation (1)), see Table 2. The increasing dopant content and heat treatment temperature results in an increasing NCs size, varying from 13 to 28 nm. Particularly, the dopant content plays a notable role in crystal growth, as it was also already observed for other GCs [35,36,37]. This effect is also related to the close relationship between the crystallization activation energy and doping level. A higher doping level implies a reduction of the crystallization activation energy which indicates that the samples with higher dopant concentration are more prone to crystallize [38]. Thus, the resulting increment of the NCs size is more intense in codoped samples.

**Figure 3:**
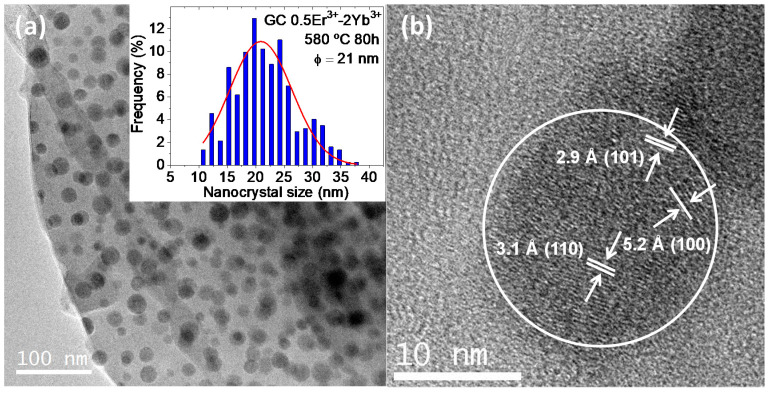
(**a**) TEM micrograph of 0.5Er^3+^–2Yb^3+^-codoped 70Si7Gd GCs treated at 580 °C-80 h. Inset, corresponding NCs size distribution; (**b**) HRTEM micrographs of the NCs with the different interplanar distances associated with the NaGdF4 structure.

HRTEM and EDX were used to get further insights into nanostructure and dopants distribution. TEM micrographs of the glasses (not shown) show the same typical phase separation droplets with the absence of any crystalline structure, from which the NCs will form after adequate heat treatment [30]. TEM micrographs of 0.5Er^3+^–2Yb^3+^-codoped 70Si7Gd GCs treated at 580 °C for 80 h show a broad crystal distribution that fits well with a pseudo-Voight function centered at 21 nm (Figure 3a, confirming the results obtained by XRD diffraction. It is worth to mention that opposite to LaF_3_ oxyfluoride NCs [39,40], the NaGdF_4_ NCs size are similar to the size of the precursor phase separation droplets, indicating that the crystallization of the phase-separation droplets occurs without further segregation into several NCs.

HRTEM micrographs of some NCs (Figure 3b) reveal the existence of a crystalline structure with different interplanar distances of 3.1, 2.9 and 5.2 Å ascribed to the (110), (101) and (100) NaGdF_4_ crystal phase crystallographic planes (JCPDS 27-0699), respectively. The values are in good agreement with the values previously reported somewhere else [41,42].

**Figure 4:**
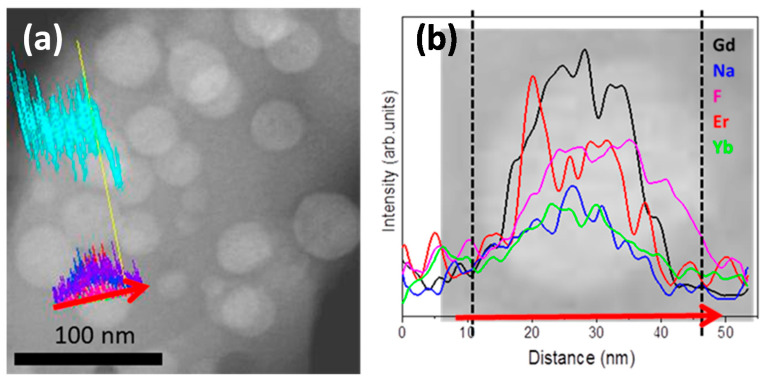
(**a**) Scanning transmission microscopy-high angle annular dark field (STEM-HAADF) image of 0.5Er^3+^–2Yb^3+^-codoped 70Si–7Gd GCs treated at 580 °C-120 h used for EDX analysis; (**b**) corresponding EDXS analysis along with the line scan spectra of one of the nanocrystals (NCs).

EDXS analysis in STEM mode was performed for 0.5Er^3+^–2Yb^3+^-codoped GCs (Figure 4) to confirm the incorporation of the RE ions into the NCs. It can be observed that Gd^3+^, Na^+^ and Yb^3+^, Er^3+^ are concentrating in the NCs, similar to previous results obtained for NaGdF_4_ GCs doped with Pr^3+^-doped and Pr^3+^–Yb^3+^-codoped GCs [30],. The EDX scan along with one of the NCs (Figure 4b) clearly shows the Gd, Na, F, Yb and Er enrichment inside the NaGdF_4_ NCs. It is worthy to note that despite the higher nominal concentration of Yb^3+^ respecting to Er^3+^ ions, these last ones are more effectively incorporated in the NCs, mainly due to the more similar ionic radius of Er^3+^ (1.062 Å) and Gd^3+^ (1.107 Å) respecting to Gd^3+^ and Yb^3+^ (1.042 Å) in 9-fold coordination [43].

### 3.2. Optical Properties

The emission spectra of the Er^3+^-doped (a) and Er^3+^–Yb^3+^ co-doped (b) glass and GCs were obtained in the 1000–1750 nm near infrared (NIR) spectral range by exciting at 974 nm of ^4^I_9/2_ (Er^3+^) and ^2^F_5/2_ (Yb^3+^) levels. The spectra show the emission band at around 1530 nm related to the ^4^I_13/2_ → ^4^I_15/2_ transition of Er^3+^ ions.

**Figure 5:**
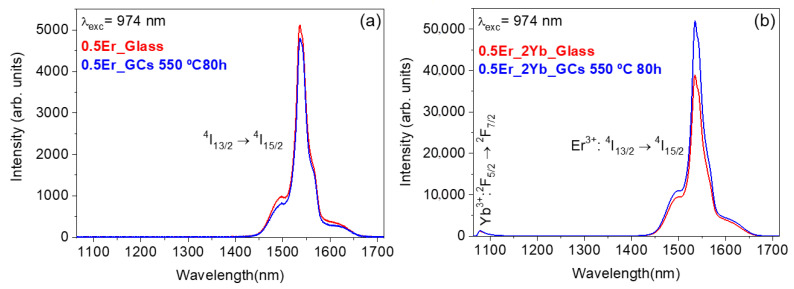
Near-infrared spectra (NIR) in the 1000–1750 nm range obtained under excitation at 980 nm of (**a**) Er^3+^-doped and (**b**) Er^3+^–Yb^3+^-codoped 70Si7Gd glass and GCs treated at 550 °C for 80 h.

**Figure 6:**
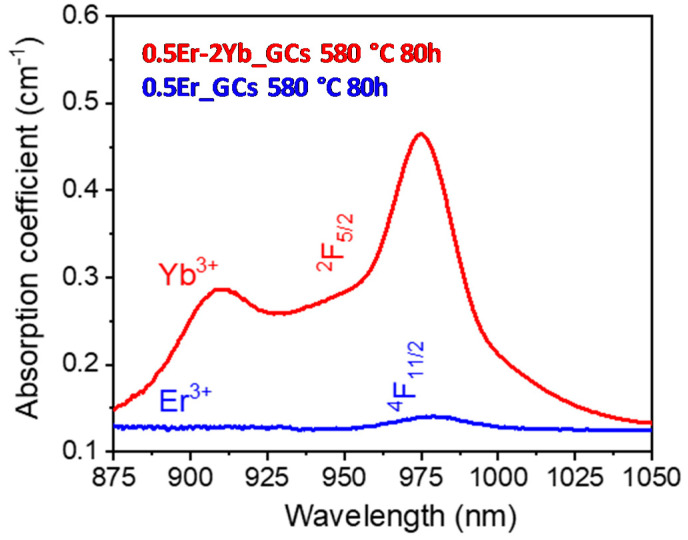
IR-absorption spectra of 0.5Er^3+^ and 0.5Er^3+^–2Yb^3+^ GCs treated at 580 °C for 80 h.

The NIR emission bands observed in Figure 5a, that corresponds to the single-doped samples present similar features with a slightly higher emission intensity in case of the glass sample. Figure 5b shows the emission spectra for the 0.5Er^3+^–2Yb^3+^-codoped glass and GCs samples. In this case, the spectra show along with the NIR emission of Er^3+^, the ^2^F_5/2_ → ^2^F_7/2_ emission corresponding to Yb^3+^ ions at around 1000 nm. The spectra of Er^3+^ ions show more intense NIR emissions, around 10 times higher, than the emissions observed before in the Er^3+^-doped GCs obtained under the same treatments. The 1.5-µm NIR emission intensity enhancement in codoped samples is mainly due to the more efficient energy transfer from Yb^3+^ to Er^3+^ ions aided by the high absorption cross-section of Yb^3+^ ion that is several times larger than the Er^3+^ one. Figure 6 shows the absorption spectra of 0.5Er^3+^ and 0.5Er^3+^–2Yb^3+^ GCs heat-treated at 580 °C for 80 h in the NIR region 875–1050 nm. It is worth to mention that despite the possible overlap between the absorption of Yb^3+^ and Er^3+^ ions in the 0.5Er^3+^–2Yb^3+^-codoped samples, it is observed that the 0.5Er^3+^–2Yb^3+^-codoped sample spectrum mainly exhibits the ^2^F_7/2_ → ^2^F_5/2_ absorption of Yb^3+^, whereas the 0.5Er^3+^-doped samples show a slighted intense and shifted peak associated with the ^4^I_15/2_ → ^4^I_11/2_ absorption level of Er^3+^ ions.

#### Up-Conversion Emission

**Figure 7:**
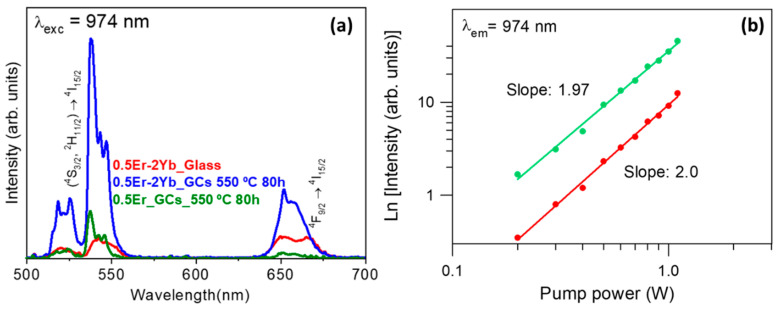
(**a**) Up-conversion emission spectra of 0.5 Er^3+^-doped and 0.5Er^3+^–2Yb^3+^-codoped 70Si7Gd glass and GCs treated at 550 °C for 80 h under excitation at 974 nm; (**b**) power dependence of UC emission intensity at 540 (green) and 650 (red) nm.

Room-temperature UC-emission spectra for glass and GCs samples obtained under IR excitation at 974 nm, are shown in Figure 7a. The selected excitation wavelength is in resonance with the ^4^I_11/2_ (Er^3+^) and ^2^F_5/2_ (Yb^3+^) levels. The spectra show the typical green emission bands associated with the (Er^3+^) (^2^H_11/2_, ^4^S_3/2_) → ^4^I_15/2_ thermalized transitions whereas the red emission band is associated with the (Er^3+^)^4^F_9/2_ → ^4^I_15/2_ transition.

As can be seen, emission spectra of 0.5Er^3+^-doped and 0.5Er^3+^–2Yb^3+^-codoped GCs exhibit characteristics features related to the RE^3+^ ions incorporation in the NCs, such as better resolved Stark emissions and higher intensities. On the other hand, a comparison between UC emissions in Er^3+^-doped and Er^3+^–Yb^3+^-codoped GCs shows that the addition of Yb^3+^ ions increases the overall UC emission, so the codoped sample exhibits a greater efficiency. This enhancement confirms the existence of an effective Yb^3+^ to Er^3+^ energy transfer process Moreover, Figure 7a also reveals that the incorporation of Yb^3+^ ions results in a color-tunable UC emission. For instance, a comparison between the red and green emissions in the 0.5Er^3+^-doped GCs and 0.5Er^3+^–2Yb^3+^-codoped ones shows that the intensity ratio decreases from 5.9 to 2.5 in codoped samples suggesting a more effective mechanism that populate the (^2^H_11/2_, ^4^S_3/2_) levels responsible of the green emissions. On the other hand, the excitation mechanisms that populate the (^2^H_11/2_, ^4^S_3/2_) and ^4^F_9/2_ levels after excitation at 974 nm were also investigated by analyzing the dependence of the UC-emission intensities at 542 and 668 nm with the lasing incident pump power. As it is well known, the UC emission intensity (I_em_) depends on the incident pump power (P_pump_) based on the relation I_em_ ∝ (P_pump_)^n^, where n is the number of photons involved in the pumping mechanism. The inset in Figure 7b shows a logarithmic plot of the integrated emission intensity of the UC green and red emissions as a function of the pump laser intensity for the 0.5Er^3+^–2Yb^3+^-codoped sample. The dependence of the intensity on the pump power indicated by the slopes in the logarithmic plots is nearly quadratic, 1.97 and 2.0 for green and red emissions, respectively. These values indicate the existence of two-photon (TP) UC process that populates levels (^2^H_11/2_, ^4^S_3/2_) and ^4^F_9/2_. This, in turn, may be associated with excited-state absorption (ESA) or energy-transfer up-conversion (ETU) [44].

**Figure 8:**
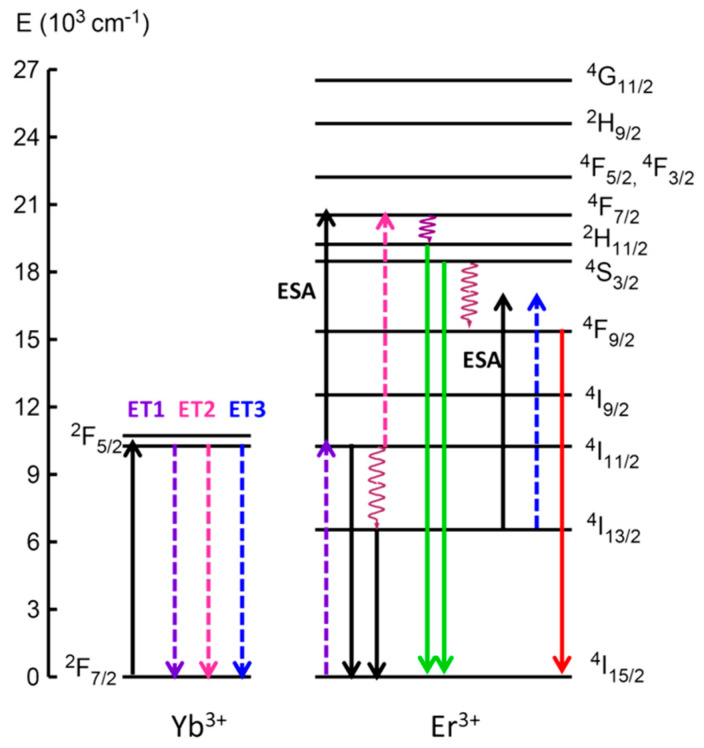
Energy level diagram of Er^3+^ and Yb^3+^ ions with the main emissions indicated by solid downwards-pointing arrows. Up-conversion (UC) mechanisms (energy transfer (ET), excited-state absorption (ESA) or energy-transfer up-conversion (ETU)) are also indicated.

Figure 8 shows the different UC mechanisms for the red and green emissions under excitation at 974 nm. When NIR photon absorption occurs at 974 nm, ground-state absorption (GSA) of Er^3+^ and Yb^3+^ ions takes place through the (^2^F_7/2_ + photon → ^2^F_5/2_) (Yb^3+^) and (^4^I_15/2_ + photon → ^4^I_11/2_) (Er^3+^) processes. As was indicated previously, Yb^3+^ ions have larger absorption cross-section ions through the (^2^F_5/2_ → ^2^F_7/2_) (Yb^3+^) transition, thus in codoped samples, this absorption is followed by an efficient Yb^3+^(^2^F_5/2_) → Er^3+^ (^4^I_11/2_) energy transfer that increases the population of the (^4^I_15/2_ → ^4^I_11/2_) (Er^3+^) ^4^I_11/2_ excited state of Er^3+^. Moreover, the Er^3+^ ions in the ^4^I_11/2_ excited level can be promoted to the ^4^F_7/2_ state by ESA of a second infrared photon ^4^I_11/2_ (Er^3+^) + photon → ^4^F_7/2_ (Er^3+^) in doped GCs or by energy-transfer from a Yb^3+^ ion (^2^F_5/2_ → ^2^F_7/2_) (Yb^3+^):(^4^I_11/2_ → ^4^F_7/2_) (Er^3+^) (ET2). Then, the ^2^H_11/2_ and ^4^S_3/2_ levels are populated by non-radiative decay from the upper ^4^F_7/2_ level. In addition, it is worthy to mention that the probabilities of an ESA or ETU process from the populated ^4^I_11/2_ level are higher in the co-doped GCs samples than those in the Er^3+^ single-doped GCs due to the ^4^I_11/2_ level population increase as a result of the energy transfer from Yb^3+^ (^2^F_5/2_) to Er^3+^ (^4^I_11/2_). Moreover, these probabilities are higher due to the reduction of the interatomic distances between Er^3+^ and Yb^3+^ ions when both are incorporated into the NCs. This reduction favors the energy transfer and consequently the enhancement of the green UC emission with respect to the red ones.

**Figure 9:**
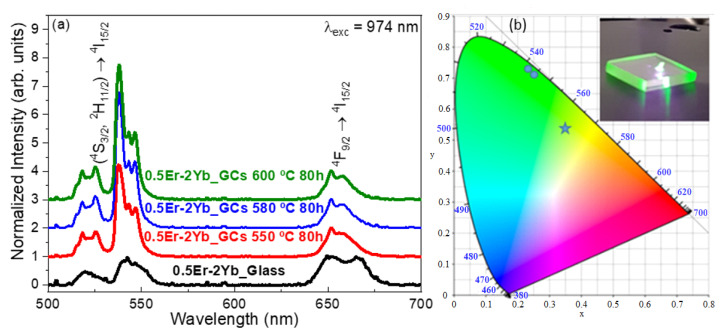
(**a**) UC-emission spectra of 0.5Er^3+^–2Yb^3+^-codoped 70Si7Gd glass and GCs treated at different temperatures for 80 h under excitation at 974 nm; (**b**) CIE-standard chromaticity diagram showing up-conversion emissions of 0.5Er^3+^–2Yb^3+^-codoped 70Si7Gd glass and GCs treated at different temperatures for 80 h under excitation at 974 nm. Inset in (**b**) picture of 0.5Er^3+^–2Yb^3+^-codoped 70Si7Gd GCs under IR excitation at 974 nm showing an efficient visible green UC luminescence.

The UC spectra of 0.5Er^3+^–2Yb^3+^-codoped 70Si7Gd glass and GCs were obtained under 974 nm excitation, see Figure 9a, in order to analyze the tuneability of the UC emissions in the GCs with the NCs size. In all cases, the spectra showed the green and red emissions corresponding to the (^4^S_3/2_, ^2^H_11/2_) → ^4^I_15/2_ and ^4^F_9/2_ → ^4^I_15/2_ transitions, respectively. The relative intensity of the green emissions strongly increased with the heat treatment temperature, opposite to that which occurs in other crystalline systems [33,45]. The maximum intensity was reached for thermal treatment at 580 °C for 80 h. For higher temperatures, there was a saturation effect, most likely related to closer interatomic distances between the RE^3+^ ions in these samples. The higher green to red UC emission ratio in co-doped 0.5Er^3+^–2Yb^3+^ 70Si7Gd GCs obtained under higher treatment temperatures may be related to the bigger NCs sizes, as it had been observed in different systems [46,47]. This effect was supported by the enhancement of the green color emission of the GCs, see inset Figure 9b. Further analysis was done in Figure 9b, that shows the CIE standard diagram with the calculated chromaticity coordinated for the precursor glass and the GCs treated at different temperatures ranging from 550 to 600 °C. The star and dots represent the CIE coordinates of precursor glass and GCs, respectively. The chromaticity coordinates change from greenish-yellow (x = 0.3403, y = 0.5561) to green (x = 0.2523, y = 0.7343) with increasing the heat treatment temperature which means that the output color could be adjusted by controlling the NCs size on GCs. This phenomenon could be explained by the fact that after heat treatments at an adequate temperature, the non-radiative relaxation process from the ^4^S_3/2_ becomes less possible, thus emitting more photons through the (^2^H_11/2_, ^4^S_3/2_) → ^4^I_15/2_ transitions, causing the enhancement of the green emission [48].

## 4. Conclusions

Transparent hexagonal β-NaGdF_4_ OxGCs were obtained using a melt-quenching process. By HRTEM measurements, it can be concluded that the crystallization occurred from (Na, Gd)- and Si phase separation droplets in the as melted glass. After adequate heat treatments of the glasses, β-NaGdF_4_ nanocrystals crystallized inside the droplets with sizes ranging from 13 to 28 nm. The increase in the dopant concentration and heat-treatment temperature provoked an increase in the NC size. The analysis performed by STEM-EDX confirmed the effective incorporation of the RE^3+^ ions in the monocrystalline phase. The incorporation in the crystalline phase was also supported by the NIR and UC spectral features of Er^3+^ ions observed in the GCs. Moreover, UC processes involving Er^3+^–Er^3+^ ions and Yb^3+^–Er^3+^ interactions ions showed a nearly quadratic dependence on the excitation power without saturation effects even in codoped samples. The color of UC emission changed from green-yellow for the glass to green for GCs by controlling the heat treatment temperature, making it possible to tune the UC emission depending on the NC size.

## Figures and Tables

**Table 1:** Glass transition temperature (T_g_), dilatometric softening temperature (T_d_) and coefficients of thermal expansion ET (α) as a function of the RE^3+^ concentration (in mol%).

	Undoped	0.5 Er^3+^	0.5 Er^3+^–2Yb^3+^
T_g_ (°C) ± 3	510	530	546
T_d_ (°C) ± 6	590	613	648
α·10^−6^ (K^−1^) ± 0.5	10.2	10.4	9.9

**Table 2:** Nanocrystals medium size, in nm, of undoped, Er^3+^-doped and Er^3+^–Yb^3+^-codoped 70Si7Gd GCs treated at different temperatures and dwelling times.

70Si7Gd	550 °C-80 h	580 °C-80 h	580 °C-120 h	600 °C-80 h
undoped	13.0 ± 1	–	17.5 ± 1	–
0.5Er^3+^	13.2 ± 1	–	20.0 ± 1	–
0.5Er^3+^–2Yb^3+^	17.0 ± 1	20.0 ± 1	23.0 ± 1	28.0 ± 1
